# Transcriptome Characterisation of the Ant *Formica exsecta* with New Insights into the Evolution of Desaturase Genes in Social Hymenoptera

**DOI:** 10.1371/journal.pone.0068200

**Published:** 2013-07-12

**Authors:** Hélène Badouin, Khalid Belkhir, Emma Gregson, Juan Galindo, Liselotte Sundström, Stephen J. Martin, Roger K. Butlin, Carole M. Smadja

**Affiliations:** 1 Centre National de la Recherche Scientifique CNRS - Institut des Sciences de l’Evolution UMR 5554, Université Montpellier 2, Montpellier, France; 2 Animal and Plant Sciences, University of Sheffield, Sheffield, United Kingdom; 3 Departamento de Bioquímica, Xenética e Inmunoloxía, Facultade de Bioloxía, Universidade de Vigo, Vigo, Spain; 4 Centre of Excellence in Biological Interactions, Department of Biosciences, University of Helsinki, Helsinki, Finland; University of Sussex, United Kingdom

## Abstract

**Background:**

Despite the recent sequencing of seven ant genomes, no genomic data are available for the genus *Formica*, an important group for the study of eusocial traits. We sequenced the transcriptome of the ant *Formica exsecta* with the 454 FLX Titanium technology from a pooled sample of workers from 70 Finnish colonies.

**Results:**

About 1,000,000 reads were obtained from a normalised cDNA library. We compared the assemblers *MIRA3.0* and *Newbler2.6* and showed that the latter performed better on this dataset due to a new option which is dedicated to improve contig formation in low depth portions of the assemblies. The 29,579 contigs represent 27 Mb. 50% showed similarity with known proteins and 25% could be assigned a category of gene ontology. We found more than 13,000 high-quality single nucleotide polymorphisms. The Δ9 desaturase gene family is an important multigene family involved in chemical communication in insects. We found six Δ9 desaturases in this *Formica exsecta* transcriptome dataset that were used to reconstruct a maximum-likelihood phylogeny of insect desaturases and to test for signatures of positive selection in this multigene family in ant lineages. We found differences with previous phylogenies of this gene family in ants, and found two clades potentially under positive selection.

**Conclusion:**

This first transcriptome reference sequence of *Formica exsecta* provided sequence and polymorphism data that will allow researchers working on *Formica* ants to develop studies to tackle the genetic basis of eusocial phenotypes. In addition, this study provided some general guidelines for *de novo* transcriptome assembly that should be useful for future transcriptome sequencing projects. Finally, we found potential signatures of positive selection in some clades of the Δ9 desaturase gene family in ants, which suggest the potential role of sequence divergence and adaptive evolution in shaping the large diversity of chemical cues in social insects.

## Introduction

Understanding the genetic basis of phenotypic adaptive traits has long been limited by the lack of genomic data. In recent years, Next Generation Sequencing (NGS) has made it possible to approach this problem by sequencing and assembly of entire genomes or transcriptomes of ecologically relevant species for which very few genomic data were previously available (reviewed in [1]).

With an impressive ecological dominance and a tremendous diversity of lifestyles [2], ants have a major impact on biodiversity and human activity. They are also a major group for the study of social evolution, among other species of Hymenoptera. The draft genomes of seven ant species, obtained using NGS technologies, have recently been published: two invasive species, *Linepithena humile* [3] and *Solenopsis invicta* [4], two leaf-cutter ant species, *Atta cephalotes* [5] and *Acromyrmex echinatior* [6], and three other model ant species *Pogonomyrmex barbatus* [7], *Camponotus floridanus* and *Harpegnathos saltator* [8]. The comparison of these genomes with those from other social and non-social insect species has revealed important insights into the evolution of ants, such as extensions of gene families involved in chemical communication (for example [3]).

However, some important sub-families and genera are not represented among those that have been sequenced (see the molecular phylogeny of Moreau [9]). One such omission is the sub-family Formicinae and the genus *Formica*, which is a key group and has been extensively studied in the context of social evolution [10]–[13]. The genus *Formica* comprises the mount-building wood ants, which dominate the ant fauna especially in boreal forest ecosystems [14]. The social organisation within the genus *Formica* is highly flexible with both inter- and intra-specific variation in queen number [15], [16]. The differences in social organisation are also often reflected in the degree of aggression exhibited towards intruders; single-queen colonies are regularly highly aggressive, whereas multi-queen colonies are often, but not always, more permissive towards intruders [17]–[19]. As a consequence, *Formica* ants are a model in studies of social organisation (e.g. [16], [20]), chemical recognition (e.g. [21]–[24]), social parasitism (exploitation of colony resources, [25]) and their role in forest ecosystems (e.g. [26]–[29]). Some populations of *Formica* in northern Europe are particularly well characterised in terms of social organisation and genetic structure, in particular *Formica exsecta* in Finland [30], [31], but little is known on the genetic basis of the traits responsible for recognition cues and social organisation. The acquisition of genomic resources for this important ant genus is necessary in order to move towards a better understanding of the mechanisms underlying various social behavioural traits, and in particular their genetic basis.

In non-model species, sequencing a transcriptome rather than a genome to get genomic data at a large scale has several advantages. It is quick, cost-effective and can thus be carried out at the level of a research group. Transcriptome sequencing can provide both expression and coding data, using RNA-seq [32]. The relatively low cost makes it possible to sequence several individuals, capturing variation in coding sequences as well as in the expression level. This approach has been successfully applied to a number of non-model species, to gain insights into polymorphism or expression information (e.g. Glanville fritillary butterfly: [33]; guppy: [34]; big sagebrush: [35]; great tit: [36]; garter snake: [37]) and to produce important genomic resources useful for various downstream approaches such as targeted re-sequencing, or microsatellite and SNP genotyping.

One application of transcriptome sequencing is to find candidate genes for particular phenotypes. In social insects, recognition of group members is necessary to direct altruist behaviour and parasitism. Nest-mate recognition involves cuticular hydrocarbons (CHC) [38]. CHC patterns differ both in the presence or absence of specific compounds or in the relative concentrations of the different components, depending on the species. In *Formica exsecta*, it has been shown that colony-specific patterns of a single type of CHC, Z9-alkenes, which varies in the distribution of C_21_–C_29_ Z9-alkenes that constitute a signal to recognise non-nest-mate ants [22], [39]. There is evidence that nest-mate recognition traits in *Formica* have both environmental and genetic components [21], [23], but their relative contributions and the identity of the genes involved remain unknown. Several gene families could be involved in the regulation of the biosynthetic pathway of Z9-alkenes. These include enzymes such as desaturases, elongases, decarboxylases and lipophorins that are carrier proteins for CHCs (e.g. [40]). The Δ9 desaturase family is a multigene family of enzymes that create a carbon-carbon double bond at the 9^th^ position from the carboxyl end of a fatty acid [41], which then undergoes several rounds of elongation and decarboxylation in order to produce a Z9-alkene. Δ9 desaturases have been shown to be involved in individual recognition, sexual selection and speciation in insects (e.g. [42]–[44]). Recently, two studies have proposed a phylogeny of this gene family in insects [3], [5]. Both studies included only one or two ant species in their dataset. Those studies identified five major clades, but some of them were not well supported. Combining desaturase sequences from multiple social and non-social insect species would improve the phylogenetic reconstruction of this gene family and allow a stronger test if desaturase genes are under positive selection [43] which might be an adaptation of sociality.

The objective of the present study was to characterise the transcriptome of *Formica exsecta* and annotate desaturase genes in this ant species to obtain extensive genomic resources for *Formica* ants. These resources will provide the means to tackle the genetic basis of important phenotypes for social insects such as CHC production/recognition and polygynous mating systems as well as look for patterns of positive selection on desaturase genes in social insects.

The *Formica exsecta* transcriptome was sequenced with 454 technology using a pooled sample of workers that represented 70 colonies (cf Material and Methods). The first step of the analysis was to assemble these sequences to accurately reconstruct the population of transcripts representing the *Formica exsecta* transcriptome. Assembling a transcriptome has its own challenges. Assembled reads, or contigs, have to represent transcripts accurately, distinguishing between alleles and paralogous genes, avoiding chimeras of several transcripts, and finding splicing variants for the same gene [45]. When there is no reference genome available, as for *Formica exsecta*, transcriptomes must be assembled *de novo*. Several *de novo* assembly programs are available, but none outperform the others significantly in transcriptome assembly [45]. To obtain a good-quality *de novo* assembly of our transcriptome, we chose to test two different assemblers with varying parameters and to compare the resulting assemblies. In the second part of this study we functionally annotated the transcriptome and analysed genetic variation in the sample. Finally, we manually annotated the Δ9 desaturase gene family in the *Formica exsecta* transcriptome dataset, reconstructed a phylogeny of insect Δ9 desaturases and searched for the signature of positive selection on those genes [46].

## Materials and Methods

### Sample Preparation and Sequencing

#### Sample collection

Ant samples were collected in summer 2008 from Joskär (59°50′44.44″N 23° 15′15.41″E), Rovholmen (59°50′15.87″N 23°15′4.28″E) and Furuskär (59°50′0.04′′N 23°15′59.90′′E N 6635900; E 2459260) islands adjacent to the Tvarminne zoological station in Hanko, Finland. The ants were collected on land belonging to the University of Helsinki field station that, via Prof. L. Sundström, gave permission for the colonies to be sampled. The ant population at this location is not considered as an endangered or protected species. To maximise information on variability among ant colonies, one ant from each colony on all three islands was chosen for the experiment (total 80 individual workers) and transferred into RNAlater (Ambion, Austin, TX, USA). The sample therefore included both social forms (i.e. single- and multi-queen colonies), and workers from single-queen colonies headed by both single- and double-mated queens.

#### RNA extraction, cDNA synthesis and normalization

Total RNA was extracted from whole bodies using TRIzol reagent (Invitrogen, Carlsbad, CA, USA) according to the manufacturer’s protocol. RNA quality and quantity was measured using the RNA 6000 Nano Chip Kit with an Agilent 2100 Bioanalyzer (Agilent Technologies, Santa Clara, CA, USA). Low-quality samples, including those with evidence of DNA contamination, were discarded (10 in total). All the samples were standardised to 200 ng/µL**^−^** ^1^, and equal volumes of 70 individual samples were combined into one single pool. The pool was DNase-treated with Turbo DNA-free (Ambion) and purified with the RNeasy Mini Kit (QIAGEN, Valencia, CA, USA) following the manufacturer’s instructions. cDNA was synthesised using the SMART procedure (Zhu et al, 2001; BD Biosciences Clontech), and then purified using the QIAquick PCR Purification Kit (QIAGEN) and finally normalised using the duplex-specific nuclease (DSN; Shagin et al, 2002) normalisation method (Zhulidov et al, 2004). cDNA synthesis and normalisation were performed by Evrogen (http://www.evrogen.com, Moscow, Russia).

#### Sequencing

The normalised cDNA pool was sequenced in one full PicoTiterPlate using the Genome Sequencer FLX Titanium Instrument from Roche Applied Science (454 Life Sciences, Branford, CT, USA) at the NERC-funded Biomolecular Analysis Facility at the University of Liverpool (http://www.nbaf.nerc.ac.uk/nbaf-liverpool).

### Transcriptome Assembly

#### Read cleaning

Reads were extracted from SFF files into FASTQ format using *sffextract* (http://bioinf.comav.upv.es/sff_extract/). Primer and adaptor sequences were masked using custom Perl scripts. Low complexity reads were screened with Tandem-repeat finder [47] (http://tandem.bu.edu/trf/trf.unix.help.html). Quality trimming was performed using *filterfastq* on a Galaxy server [48]: parts of the sequences with the lowest quality scores (<25), mostly at 3′ ends, were removed as well as sequences shorter than 50 bp. Poly-A tails were screened with *trimest* from the *EMBOSS* package [49] (http://emboss.sourceforge.net/apps/release/6.1/emboss/apps/trimest.html) and masked in the fastq file with Biopython. Contaminant or unwanted sequences were identified by searching (*blastn* [50]) raw reads against (i) the *Wolbachia pipientis* genome (e-value cut-off of 10e-45, [51]) (ii) rRNA coding genes of the *Formica* genus (e-value cut-off of 10e-45 [52], http://www.arb-silva.de/). These reads were removed from the data set using a Biopython script. The resulting FASTQ file was used as input for *de novo* assemblies.

#### De novo assembly

To optimise the quality of the *de novo* transcriptome assembly, we compared two different assembler programs: *Newbler 2.6* (Roche), recommended in Kumar and Blaxter [45] for transcriptome assembly, and *MIRA 3.0* [53]. We tested two main assembly parameters for each program: minimum percentage identities from 85 to 95%, and minimum overlap length of 20 bp and 40 bp for *MIRA*, and 40 and 60 bp for *Newbler*. The “-cdna” mode was used for *Newbler*. We also tested a new option of *Newbler 2.6*, the *–urt* option, which is dedicated to improve contig formation in low depth portions of the assemblies. Assembly metrics (number of contigs, the total length of assembly, the average contig length, and the number of singletons – i.e. the number of unassembled reads) were computed on Galaxy. Qualitative assessment of the different assemblies was performed by measuring the coverage of a set of 50 known transcripts [32] already annotated in the genus *Formica* or its closely related ant species *Camponotus floridanus*. For each gene, the total percentage of its length covered by the assembly (completeness) and the maximum percentage covered by a single sequence (contiguity) were computed [32]. BLAST of assemblies against the test transcripts, qualitative assessment of coverage and measures of contiguity and completeness were carried out on a Galaxy server. The final *de novo* assembly was chosen based on basic assembly metrics and performance in terms of completeness and contiguity.

#### Clustering

*Newbler* is sometimes too conservative in its method for assembling reads. This can cause alleles to be separated into different contigs, even after *Newbler* has performed a clustering step and grouped presumed alleles in “isotigs” [37]. When several contigs blasting to the same test transcript shared a high percentage similarity, we hypothesised that they were redundant contigs for the same transcript. We evaluated redundancy qualitatively in the *trackster* viewer on a Galaxy server, and empirically determined a minimum percentage identity of 98% to cluster contigs. Contigs of *Newbler* assemblies were clustered with *uclust* [54] on Galaxy, with the minimum identity set to 0.98 and otherwise default parameters.

#### Mapping

We used the recently available genome of *C. floridanus* to run a mapping assembly where *de novo* contigs were mapped onto *C. floridanus* predicted transcripts with *bwa* [55] (http://bio-bwa.sourceforge.net/), using standard parameters.

### Transcriptome Annotation

#### Similarity annotation

Contigs and singletons of the best-quality assembly were searched against the non-redundant (nr) protein database of NCBI with an e-value cut-off of 10e-5 (*blastx* program). The latest versions of ant protein datasets were downloaded from the Hymenoptera database [56] (version 3.8 for *Acromymex echinatior*, 1.2 for *Atta cephalotes*, 3.3 for *C. floridanus*, 3.3 for *Harpegnathos saltator*, 1.2 for *Pogonomyrmex barbatus*, 1.2 for *Linepithema humile* and 2.3 for *Solenopsis invicta*) [3], [5]–[8]. Sequences that did not have any match in the nr database were blasted against the combined ant datasets with an e-value cut-off of 10e-5.

#### Gene Ontology (GO) annotation

Contigs and singletons of the best-quality assembly were annotated with gene ontology terms with *Blast2GO* [57] online or pipeline version (http://www.blast2go.com/b2glaunch/resources), using *blastx* reports and Blast2GO default e-value cut-off (10e-6). Sequences were annotated for enzyme codes (EC) and KEGG (Kyoto Encyclopedia of Genes and Genomes) biochemical pathways [58] with online *Blast2GO*. A GO Slim analysis was run with the generic protocol (generic.obo) to obtain a simplified visualisation of functional annotations. We made a qualitative comparison between the results of the GO Slim analysis and those performed for other insect species, namely *L. humile* [3], *Nasonia vitripennis* [3], *Tribolium castaneum* [59], and *Drosophila melanogaster* [3]. We used Table S6 of the genome analysis of *L. humile* [3], which compares the percentage of genes matching a set of GO terms.

#### Prediction of Open Reading Frames (ORF)

For sequences with a significant *blastx* hit (10e-8), ORFs were predicted with the *prot4EST* pipeline [60] version 2.2, modified to accept xml BLAST input files. For the remaining sequences, *ESTScan2* [61] was used to detect ORFs based on the bias in codon usage. The species-specific matrix, used in *ESTScan2* to represent codon usage in *Formica exsecta*, was built by *Prot4EST3.1b* from the output of *blastx* searches. The sequences without any *ESTScan* prediction were passed to *getorf* (EMBOSS package [49], http://emboss.sourceforge.net/). A six-frame translation was performed and the longest ORF between two stop codons was kept, with a minimum length of 30 bp.

### Analysis of Polymorphism

#### SNP calling and filtering

Reads used for building contigs in the assembly were re-mapped to the *de novo* assembly using *bwa* [55] (http://bio- bwa.sourceforge.net/). The alignments were processed with *samtools* [62] (http://samtools.sourceforge.net/) and SNPs were called using *VarScan* [63], a tool that is adapted to SNP calling from pooled samples. By default, *VarScan* calls SNPs at position with at least 8X coverage, 2 reads supporting the variant allele, a minimum frequency of 5% for the variant and a minimum base-calling quality of 15. To be even more conservative and further minimise putative sequencing errors, only SNPs with at least 3 reads supporting the variant allele were kept.

#### Highly variable contigs

The degree of variability of a given transcript can indicate selection acting on the gene. We therefore computed the Pearson’s correlation between the number of SNPs and the contig length and its p-value, as well as the distribution of SNP number per contig to identify highly variable contigs in the transcriptome dataset.

### Desaturase Gene Family Annotation and Analysis

#### Annotation

We used sequence similarity with other insect sequences to identify desaturases in the *F. exsecta* transcriptome data set using *blastx.* The **Δ**9 desaturases of *L. humile* had been manually annotated in [3], *Drosophila melanogaster* desaturases were retrieved from flybase [64] (CG9747, CG9743, desat1, desat2, desatF, CG15531 and GC8630) and we manually annotated desaturases from the closely related ant species *C. floridanus* using available CDS predictions [56] (http://www.antgenomes.org/). Annotations were manually curated and final annotations confirmed with reciprocal BLAST searches against protein databases. Desaturase sequences homologous to sub-families other than the **Δ**9 family were not considered in subsequent analyses.

#### Gene phylogeny

Phylogenetic analyses were conducted on most of the **Δ**9 desaturase genes found in *F. exsecta* as well as other representative insect **Δ**9 desaturases. In addition to *L. humile, C. floridanus* and *D. melanogaster* sequences already retrieved for the annotation step, desaturase sequences from five other ant species (*S. invicta, A. echinatior, A. cephalotes, P. barbatus, H. saltator*) were retrieved by searching with *blastp* the combined dataset of expressed and predicted ant proteins [56] using *L. humile* desaturases as queries. The sequence set was also completed with desaturases from three other hymenopteran species (*Apis mellifera, Apis floridanus* and *Nasonia vitripennis*) searched in the hymenopteragenome dataset, as well as other hymenopteran and non-hymenopteran insect **Δ**9 desaturases (*Bombus terrestris*, *B. impatiens, Megachile rotundata, Acyrthosiphon pisum, Tribolium castaneum*, *Bombyx mori*, *Anopheles gambiae*) retrieved using *D. melanogaster* desaturases as queries for *blastp* searches against the NCBI non-redundant protein database. Pseudogenes were excluded from the analysis. Partially annotated genes were excluded only when they were very short compared to other sequences. Accession numbers for all insect desaturases used in this study are provided in Table S1.

The amino acid sequences of 197 homologous genes were aligned using *MAFFT* v6 [65]. *Gblocks* [66] with low stringency parameter settings, and *Guidance* [67], were used to screen positions that were poorly aligned. We compared the results of those two programs to manually curate the alignment in *Jalview* [68]. This resulted in a final trimmed dataset comprising 282 amino acid positions. LG+G was found to be the best evolutionary model for the dataset as determined by *ProtTest* [69]. A nucleotide alignment guided by the amino-acid alignment was obtained with *translatorX*. The best evolutionary model (GTR+I+G) was inferred with *jModelTest* [70]. According to the best models for nucleotide and amino acid alignments, maximum likelihood trees were reconstructed using *RAxML* v7.0.4 [71], and nodal support values were obtained by a 500-replicate rapid bootstrap analysis.

#### Selection analysis

To investigate difference in selective pressures among **Δ**9 desaturase genes of social Hymenoptera, sequences from A. mellifera, A. florea, B. impatiens, B. terrestris, L. humile, P. barbatus, S. invicta, A. echinatior, A. cephalotes, C. floridanus, H. saltator and F. exsecta were re-aligned independently and a maximum likelihood tree was obtained with the same methodology as for the general insect tree. Tests for variation in selective pressures and for positive selection were performed using the codeml program of the PAML package [46] which estimates, by a maximum likelihood method, ω ratios of the normalised non-synonymous substitution rate (dN) to the normalised synonymous substitution rate (dS). ω>1 is considered to be evidence of positive selection for amino acid replacements, whereas ω<1 indicates purifying selection. The comparison between models was assessed using Likelihood-Ratio Tests (LRTs) for hierarchical models [72]. Only sequences from species of Hymenoptera (cf. phylogenetic analyses) were chosen for selection analyses. We aligned all hymenopteran protein sequences using MAFFT v6 and the nucleotide sequence alignment was guided by the protein sequence alignment with translator [73].

We first estimated dS for all branches with a free ratio model (M0) and computed the mean synonymous distance for the different clades. To avoid saturation, only clades with a mean synonymous distance less than 1 were subsequently considered for selection analysis [72], [74], [75].

We then tested for variation in selective pressures among major desaturase clades using branch-specific models [76]. By default, branches had the same background ω, ω_0_, and only branches under study (foreground branches, here the major desaturase clades with a dS<1) were allowed to have a ω value different to ω_0_. To test for some differences in selective pressures among these desaturase clades, the branch-specific method that we applied here compared the following two models: the null model fixes a single ω for all foreground branches, whereas the alternative model allows the ω ratio to vary among foreground branches in the phylogeny [74], [77]. Thus, a significantly higher likelihood of the alternative model than that of the null model indicates variation in selective pressures among branches [74], [77].

To test for positive selection at specific sites in the alignment, we compared the M8a-M8 models with a likelihood-ratio test (LRTs) for hierarchical models [72], a test which has been shown to be conservative and robust against violations of various model assumptions, and to produce fewer false positives than the M7–M8 comparison [78], [79]. We applied this test to clades in which ω was greater than the background ω_0_ in the branch-specific test. To further confirm our results, we additionally used an LRT comparing M1a (Nearly Neutral) and M2a (Selection) models, which has been shown to be one of the most conservative tests for selection (e.g. [79], [80]). For both M8a–M8 and M1a–M2a comparisons, we used degrees of freedom, df = 2. Only in cases where the LRT was significant, we used the Bayes empirical Bayes (BEB) procedure to calculate the PPs to identify sites under positive selection [80]. In clades showing evidence of positive selection at some sites, we used branch models and/or branch-site models to test whether positive selection affected specific duplications events. For branch-site analyses, we compared an alternative model where a subset of sites in the foreground branches have ω2>1, with a null model where ω2 = 1 [76]. A correction for multiple tests was applied to the site model comparisons following the method of Benjamini and Hochberg [81].

### Data Release

Raw data were released on the short read archive (SRA) of NCBI as two sff files under accession number SRR837406 and SRR848070. The assembled transcriptome, gene ontology annotation and SNP dataset are available on the antgenome database (http://www.antgenomes.org/).

## Results and Discussion

### Transcriptome Sequencing and Assembly

#### Sequencing statistics and read cleaning

Sequencing of the transcriptome of *F. exsecta* produced 1,051,346 raw reads, with a mean length of 374.7 bp, for a total length of 390 Mb and a GC content of 35.4%. We removed 333 reads blasting to the *Wolbachia pipientis* genome [51], 101 rRNA reads and 13,992 chimeric, low-quality or short reads (<30 bp). After read cleaning, the dataset included 1,036,920 reads with a mean length of 315.5 bp (total: 327 Mb).

#### Comparison of Newbler2.6 and MIRA 3.0

With default parameters, *MIRA* produced more (32,929 versus 17,955) and shorter (809 versus 893 bp) contigs than *Newbler*, which is consistent with a recent comparative study showing that *MIRA* is more conservative in its way of assembling reads [82]. Moreover, the total length of the alignment was 26.6 Mb with *MIRA* versus 16 Mb with *Newbler* (Table S2). When the *Newbler* option *-urt* is activated, *Newbler* builds contigs from slightly overlapping reads and extends contigs to low-coverage extremities. This option increased the total length of assembly from 16 Mb to 23.2 Mb, closer to the total length of *MIRA* assemblies, increased the number of contigs to 32,576 (Table S3) and decreased the number of singletons to 3,769. The average contig length of this assembly was reduced to 722 bp, due to the formation of many new short contigs. Hence, activation the -*urt* option greatly improved the quality of the transcriptome assembled and made *Newbler* outperform *MIRA* on our dataset.

When evaluating the quality of *MIRA* and *Newbler* assemblies using 50 test genes, we found that 15 transcripts annotated in species other than *F. exsecta* were never covered by any assembly. However, we never observed a gene covered by a contig with one assembler and not the other. Completeness, computed on a *MIRA* and a *Newbler* assembly with comparable parameters (90% minimum identity and 40 bp minimum overlap length, *-urt* option improving coverage and additional clustering for *Newbler*), was 81% for *MIRA* against 84% for *Newbler* (Table S4B). Contiguity, which was computed for each test transcript on assemblies obtained with different combinations of assembly parameters, ranged from 55% to 67.9% with *MIRA* and from 72.0% to 74.4% with *Newbler* (Table S4A). Thus, contiguity was higher with *Newbler 2.6* and was also more robust to changes in assembly parameters. This approach consisting in selecting a set of known test transcripts to assess the quality of a transcriptome assembly [32] therefore allowed us to discriminate between assemblies that were similar in terms of classic metrics (number of contigs, total assembly length, average contig length and number of singletons) and demonstrated its value for choosing among different possible assemblies in other species.

#### Clustering and mapping

It had been reported that *Newbler* creates redundant contigs [37]. This is confirmed in our results: *Newbler* performs a clustering step, but we still found redundancy in our dataset. When clustering *Newbler* contigs to avoid redundancy, we reduced the number of contigs from 32,580 to 26,791, while conserving completeness and contiguity values. The additional clustering step we performed was therefore able to reduce redundancy without affecting performance of the assembly on our test transcripts. The mapping strategy against the *C. floridanus* reference genome did not result in a high-quality assembled dataset, with many reads that did not map to the *C. floridanus* reference sequences. The distance between the two genera, *Camponotus* and *Formica*, probably explains this result: they are likely to have diverged in the late Cretaceous, the most intense period of ant radiation [9]. Moreover, alternative splice variants might have also complicated the mapping of transcribed sequences against *C. floridanus* genomic sequences.

In another study comparing different assemblers, Kumar and Blaxter [45] had found that *MIRA 3.0* and *Newbler 2.5* had similar performances. More recently, Mundry et al [82] found that these two assemblers outperformed other programs for 454 transcriptome assembly, *Newbler 2.5* assembling reads in a more liberal way than *MIRA 3.0*. Here, our study suggests that *Newbler 2.6* can outperform *MIRA* under certain conditions and provides insight and guidance on how to optimise *Newbler* assembly of 454 transcriptome data.

We retained the *Newbler* assembly obtained with the parameter values of 90% overlap identity over a 40 bp minimum overlap length and the *–urt* option, followed by a clustering step, as the optimal assembly for subsequent analyses (Table 1). In this *de novo* assembly, 99.6% of the reads were assembled, for a total length of 27 Mb of sequences. This final assembly of 454 high quality reads resulted in a mean coverage of 11X and 26,791 contigs, while 3,768 reads remained as singletons. Contigs ranged from 40 to 9,700 bp in size with a mean contig length of 1,008 bp. The distributions of contig and singleton lengths are shown in Figure S1. These figures are in the range of values for similar studies reviewed by Fraser et al [34].

**Table 1:** Assembly statistics for the transcriptome of *Formica exsecta*.

Numberof contigs	Cumulated lengthof contigs (Mb)	Maximum contiglength (bp)	Average contiglength (bp)	Number ofsingletons	Average depthof coverage
26,791	27	9,700	1,008	3,769	11X

### Transcriptome Functional Annotation

#### Similarity search annotation

**Figure 1:**
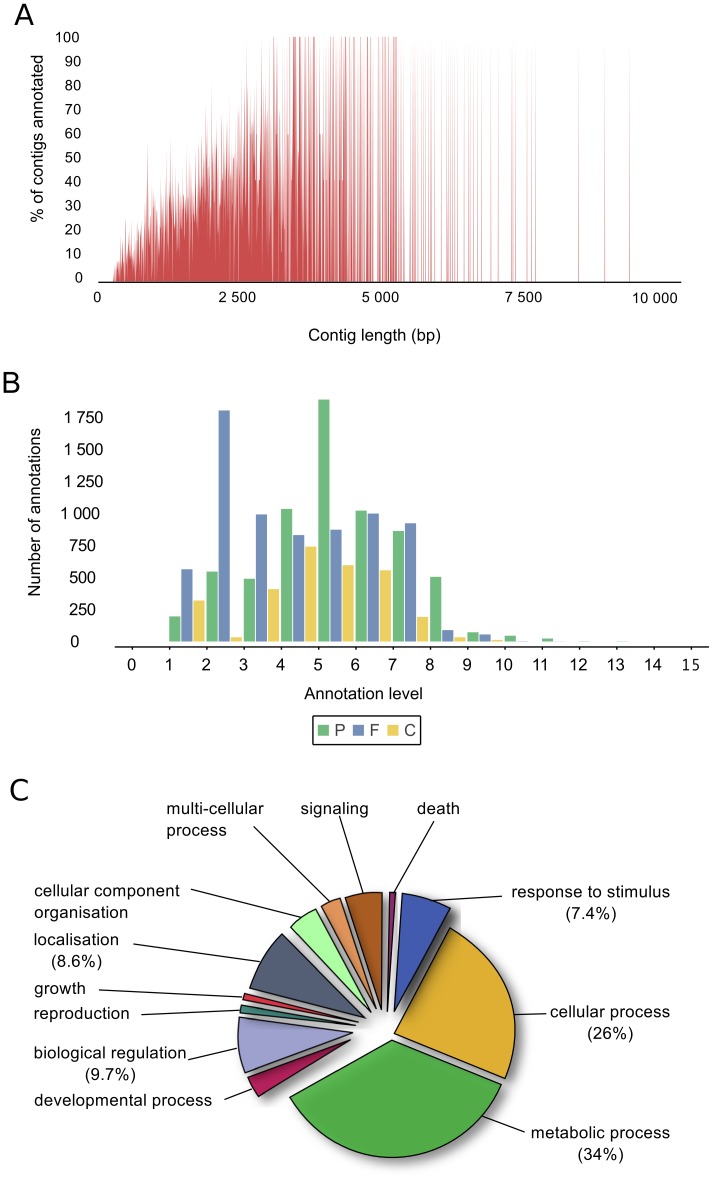
Gene ontology (GO) annotation of the transcriptome of *Formica exsecta*. A – Number of sequences associated with at least one GO term in relation to sequence length. B – Distribution of hierarchical level of GO annotations. For each annotation level, the number of sequences mapping to a term is shown for biological process (P, in green), molecular function (F, in blue) or cellular localisation (C, in yellow). C – Proportions of annotations corresponding to the high-level biological processes in the transcriptome of *Formica exsecta*. Terms correspond to level 2 of the GO hierarchy.

Among the 26,791 contigs and 3,769 singletons initially used for the *blastx* search against the NCBI *nr* protein database, 12,668 (41%) sequences had matches, including 8,269 unique hits, a figure in the range of those obtained for other non-model species. The most represented taxa for the best hit of each match were two hymenopteran species: *Apis mellifera* with 7,438 hits and *Nasonia vitripennis* with 3,001 hits. The annotation of the ant genomes is still ongoing so new gene annotations are released on the Hymenoptera database before being released on NCBI. When blasting the remaining 17,891 sequences against the predicted protein data sets of seven ant species, we identified 3,052 additional sequences with matches, highlighting the importance of annotated genomic data from closely related taxa. Indeed, ants and wasps diverged during the Jurassic, around 185 My ago, while the ant genera *Formica* and *Camponotus* diverged only in the late Cretaceous. In total, 15,720 (51.4%) sequences were annotated. 14,839 (48.5%) sequences did not show significant similarity to any protein in the databases and could not be annotated. There was a positive relationship between sequence length and the percentage of annotated sequences (Figure 1A).

#### Gene Ontology assignment

A total of 7,100 sequences (23.3%) could be assigned with one or more gene ontologies using Blast2GO (e-value cut-off 10e-6), which is consistent with values from similar studies. Interestingly, Fraser et al [34] obtained similar annotation rates for a similar minimum divergence time (70 My) with a sequenced species. Of those sequences, 4,878 (32.9%) were annotated with a molecular function, 3,100 (22.2%) with a biological process and 2,045 (14.5%) with a cellular component. The distribution of annotated contigs under different GO levels of each category (Figure 1B) showed a concentration in levels 5–8, 3–7 and 4–7, respectively, for biological process, molecular function, and cellular component, indicating a good accuracy of annotation. The most highly represented GO terms for a molecular function were binding (52.4%) and catalytic activities (35.3%) and the most highly represented GO terms for a biological process were metabolic process, cellular process, localisation and biological regulation (Figure 1C).

For GO terms potentially linked to the metabolism of cuticular hydrocarbons and chemoreception, we found 41 sequences that had an odorant binding function and 33 sequences with an olfactory receptor putative activity. To find sequences potentially involved in the metabolism of cuticular hydrocarbons, sequences were annotated with enzyme codes (EC). We could identify an enzyme code to 1,363 sequences. The KEGG database was used to link enzyme codes with metabolic pathways. The following enzymes implicated in the elongation of fatty acids were found: long-chain-enoyl-CoA hydratase, paltiptoyl hydrolase, enoyl-CoA hydratase and 3-hydroxyacyl-CoA dehydrogenase. We identified eight sequences involved in the biosynthesis of unsaturated fatty acids that corresponded to the enzymes stearoyl-CoA 9-desaturase, acyl-CoA oxidase and enoyl-CoA hydratase.

When comparing results of the GO Slim analysis for *F. exsecta* to those performed for other insects, we showed that some categories of GO terms have a similar representation in *F. exsecta* and other insect species. For example, 10.9% of sequences in *F. exsecta* could be associated with a biological process, versus about 11.5% in *L. humile*, *N. vitripennis* and *A. mellifera*; 14.6% of sequences in *F. exsecta* could be associated with a molecular function, versus around 16% in other hymenopteran species and 11.08% in *D. melanogaster*; and around 10% of sequences could be associated with a catalytic activity in *F. exsecta*, *L. humile*, *N. vitripennis* and *D. melanogaster*. In contrast, some GO terms show significant differences with an overrepresentation in *F. exsecta*, such as the cellular localisation function (7.2% in *F. exsecta* versus 3.5 to 4% in the other species) and the binding function (10% in *F. exsecta* versus 2–3% in other species). We also looked at the GO Slim analysis carried out on the transcriptome of *T. castaneum* and showed very similar representation of GO terms (35% of annotations for a molecular function of level 3 in *F. exsecta* corresponded to a catalytic activity, versus 36% in *T. castaneum*; 52% of annotations for a molecular function of level 3 in *F. exsecta* corresponded to a binding activity, versus 41% in *T. castaneum*). Overall, the percentage of sequences associated with the different high-level GO terms in *F. exsecta* was consistent with findings in other insect species, suggesting that our 454 dataset is a good representation of the *F. exsecta* transcriptome.

#### Open reading frame prediction

For sequences that had matches against the *nr* database with an e-value lower than 10e-8, high scoring segments for the same hit protein were joined and extended. This step predicted 12,329 ORFs with a minimum length of 30 bp. For those sequences that didn’t meet the e-value cut-off (10e-8) or had no BLAST hit, *ESTScan* predicted 2,715 (14.8%) new ORFs. For the remaining sequences, a six- frame translation was carried out and the longest open reading frame was kept. As a result of this stepwise procedure, we could assign 99.9% of contigs and singletons with an ORF of at least 30 bp. Among these, 28,993 (94.5%) sequences had an ORF of at least 100 bp. Many studies discard ORFs shorter than 300 bp. However, it has been shown that a few real proteins are included in ORFs from 150 to 300 bp [83]. The ORF mean length was 452 bp. Only 451 of the identified ORFs were complete (starting with a start codon and ending with a stop codon), while 30,108 ORFs were partial, among which 2,068 were truncated at the 3′ terminus, 4,296 at the 5′ terminus and the rest at both ends. This shows the limit of 454 sequencing of cDNA to produce full-length transcripts, although truncation at 5′ termini could also result from the SMART cDNA synthesis procedure or biases in normalisation. In particular, the quality of sequencing drops at the extremities, which makes it difficult to get complete transcripts.

### SNP Discovery and Analysis

With default parameter values, 34,701 SNPs were predicted. As SNP calling is subject to two main sources of error: sequencing errors (more than 1% in 454) and incorrect mapping of reads to the reference, we applied a stringent filtering procedure (see Material and Methods) and after filtering, a total of 13,567 sites (39%) were considered as high-quality SNPs and thus were kept. In this *F. exsecta* transcriptome dataset, 4,747 (17.7%) contigs had at least one SNP. The mean number of SNPs per contig was 0.5. There were 0.5 SNPs per 1,000 bp on average. We found a positive correlation between the number of SNPs and the length of the contig (y = 0.0048x, r = 0.91, p-value<0.05) (Figure S2). Some of the most polymorphic genes had precise GO annotations: a fatty acid synthase, a deoxythymidylate kinase, a galactosyltransferase, a chaperone for superoxide dismutase and a heat shock protein.

Although one needs to keep in mind limitations of SNP calling from pooled samples and possible complications arising from duplicated regions of the genome (e.g. multigene families), our study identified highly variable contigs that are potentially under diversifying selection and provided a unique set of SNP markers offering new opportunities to design SNP genotyping arrays and investigate genetic variation at candidate genes involved in chemical recognition and social behaviours across different *Formica* species, populations, colonies or castes.

### Desaturase Gene Family Annotation and Analysis

#### Annotation

BLAST searches with *blastx* identified 13 desaturase sequences in the transcriptome of *F. exsecta*. Among these sequences, three did not belong to the **Δ**9 desaturase family, and four were short fragments. One long N-terminal and C-terminal truncated sequence and five complete sequences were retained for subsequent analyses (sequence information in File S1). Although we cannot exclude the possibility that there are other Δ9 desaturases in the *F. exsecta* genome, such as close paralogs grouped as alleles, rare variants we might not have caught in the sequencing experiment or genes not expressed in the adult worker individuals studied, it seems unlikely that the Δ9 desaturases underwent a large expansion in *F. exsecta*. *C. floridanus*, the closest sequenced ant species, does not show any large expansion such as those seen in *L. humile* or *S. invicta* either.

BLAST analyses of the *F. exsecta* **Δ**9 desaturase genes against *D. melanogaster* sequences found that three were most similar to the *D. melanogaster desat1* gene (70.5%, 55.2% and 54.5% of identity), two to the *D. melanogaster* gene CG9747 (59.5% and 59.1% of identity), one to the *D. melanogaster* gene CG15531 (36.7% of identity), and none was found to be most similar to *D. melanogaster desat2*, *desatF*, CG9743 or CG8630. Four small fragments of desaturases were found to be most similar to *desat1* and one to *desat2*.

#### Gene phylogeny

**Figure 2:**
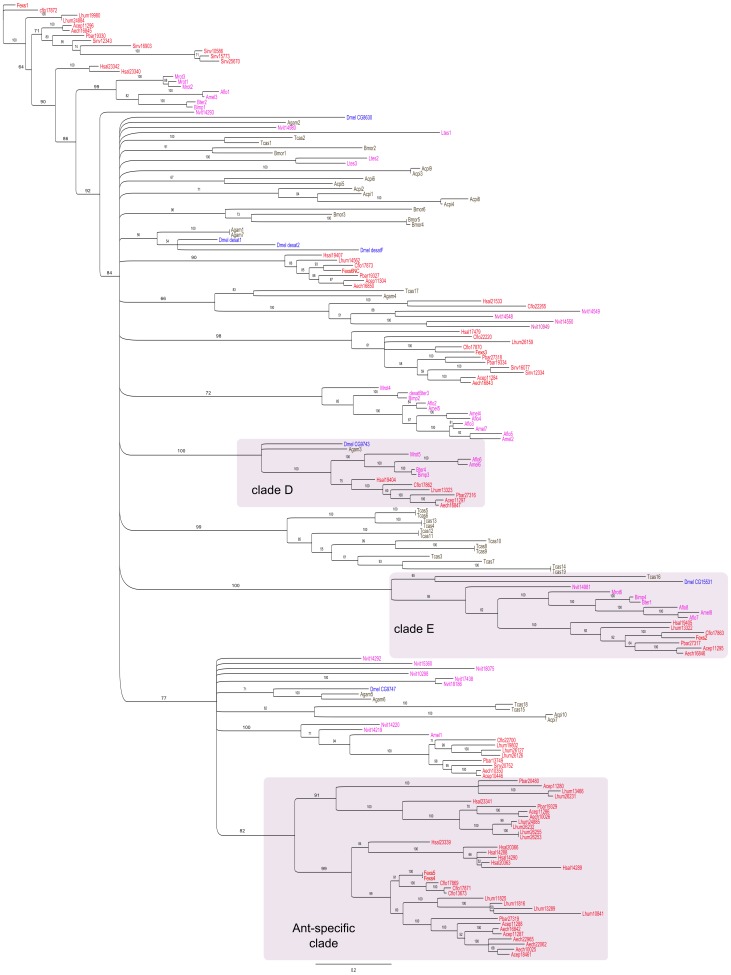
Maximum likelihood phylogeny of insect Δ9 desaturases. Bootstrap values greater than 50 are shown on the tree. Branches with a bootstrap value under 50 are collapsed to show unresolved parts of the tree as polytomies and to underline supported clades (among which are clades D, E and an ant-specific clade, emphasised in light purple shading). Desaturase sequences from *Drosophila melanogaster* are shown in blue (e.g. Dmel CG9747, Dmel *desatF*). Sequences from other non-Hymenoptera insects are shown in black (Tcas: *Tribolium castaneum*; Agam: *Anopheles gambiae*; Bmor: *Bombyx mori*; Acpi: *Acyrthosiphon pisum*). Non-ant Hymenoptera sequences are shown in pink (Nvit: *Nasonia vitripennis*; Bter: *Bombus terrestris*; Bimp: *Bombus impatiens*; Amel: *Apis mellifera*; Aflo: *Apis floridanus*; Mrot: *Megachile rotundata*). Desaturase ant sequences are shown in red (Hsal: *Harpegnathos saltator*; Lhum: *Linepithema humile*; Cflo: *Camponotus floridanus;* Fexs: *Formica exsecta*; Pbar: *Pogonomyrmex barbatus*; Acep: *Atta cephalotes*; Aech: *Acromymex echinatior;* Sinv: *Solenopsis invicta*).

An alignment of 282 codons was obtained and used to reconstruct a maximum likelihood phylogeny of the **Δ**9 desaturase gene family in insects, with a focus on Hymenoptera and in particular ant species. The tree obtained from the alignment of all insect desaturases selected for this analysis is shown in Figure 2. Branches with a bootstrap support value under 50 were collapsed. Some parts of the tree were not well resolved, but several robust clades appeared: the clades of sequences orthologous to CG9743 (clade D) and CG15531 (clade E), respectively, were well supported. *F. exsecta* was present in clade E and absent in clade D but genome data would be needed to confirm this absence. These clades were also well supported in previous studies [3], [5]. However, in contrast to studies [3] and [5], clade A, corresponding to sequences similar to the desaturases of *D. melanogaster desat1*, *desat2*, *desatF*, and CG8630, and clade B, corresponding to sequences similar to CG9747, were not supported in our analysis. Nevertheless, a subset of the sequences primarily assigned to clade A and which includes a desaturase of *F. exsecta*, formed a hymenopteran-specific clade with high bootstrap support. The moderately supported clade with sequences orthologous to CG9747 showed a large polytomy at its base, and included a well-supported and large ant-specific clade with important expansions in several ant species. This clade is an important candidate for involvement in the evolution of chemical signaling function after the ant radiation. *F. exsecta* showed only one duplication in this clade, but recent duplication could have been missed as very close sequences can be mistaken as allelic differences.

Discrepancies between our desaturase phylogeny and the ones previously found by [3] and [5] could have a basis in methodological differences. Although we used very similar methods (alignment and phylogenetic programs), our dataset comprised more Hymenoptera species than in the previous studies and we used a different substitution model from [3]. This model was not yet implemented in the version of RAxML that they used, and proved to be the best evolutionary model for our dataset. However, with a more stringent curation of the alignment and the same evolutionary model as in [3], we retrieved the same major differences (clades A and B not supported). This suggests that increasing the number of sequences and species might have modified the reconstruction and revealed clades whose support was not robust.

#### Selection analyses

**Figure 3:**
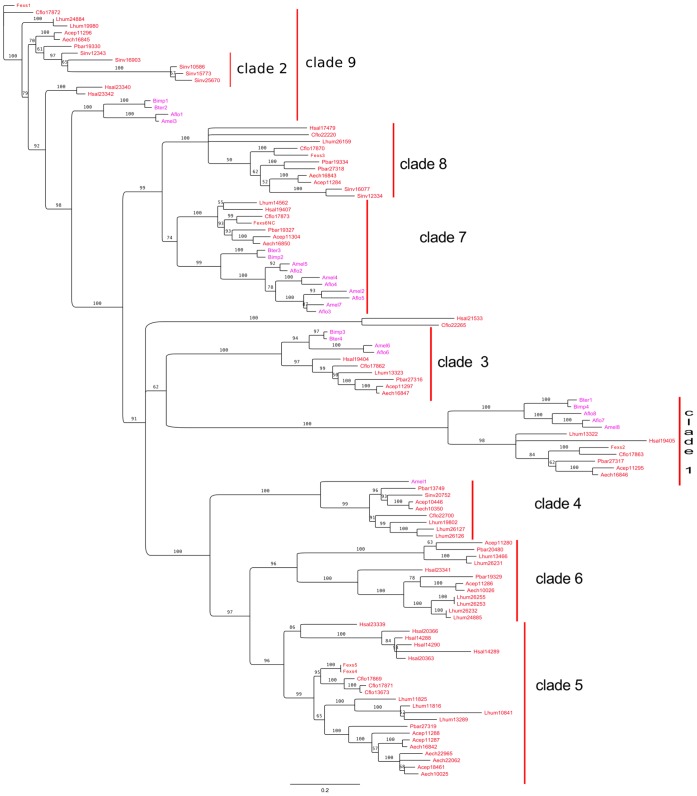
Maximum likelihood phylogeny of Δ9 desaturases in only social Hymenoptera. Bootstrap values greater than 50 are shown on the tree. Branches with a bootstrap value under 50 are collapsed to show unresolved parts of the tree and underline supported clades. Clades shown with vertical red bars correspond to well-supported clades chosen to perform selection analysis. Non-ant social insect desaturase sequences are shown in pink (Bter: *Bombus terrestris*; Bimp: *Bombus impatiens*; Amel: *Apis mellifera*; Aflo: *Apis floridanus*). Ant desaturase sequences are shown in red (Hsal: *Harpegnathos saltator;* Lhum: *Linepithema humile*; Cflo: *Camponotus floridanus;* Fexs: *Formica exsecta*; Pbar: *Pogonomyrmex barbatus*; Acep: *Atta cephalotes*; Aech: *Acromymex echinatior;* Sinv: *Solenopsis invicta*).

**Figure 4:**
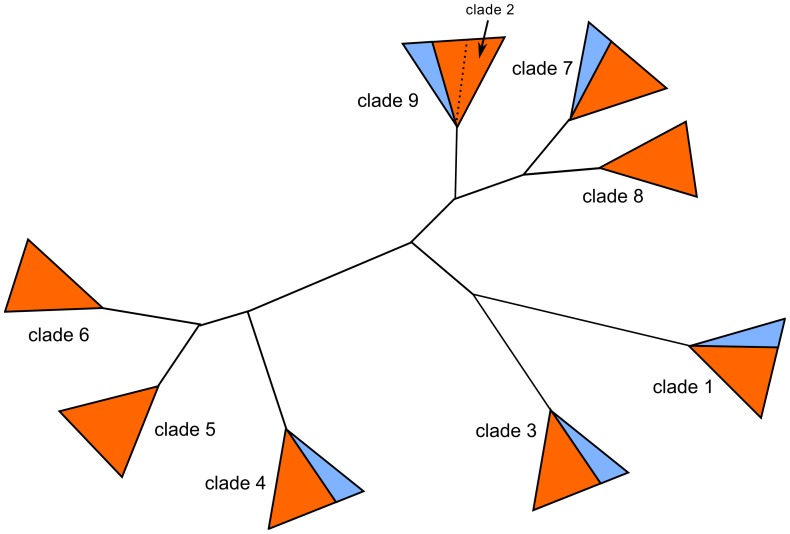
Star representation of the phylogeny of Δ9 desaturases in social Hymenoptera highlighting the clades that have been chosen for selection analyses (1 to 9). Ant-specific foreground branches are coloured in orange. Non-ant sequences are indicated in blue.

As we found several robust clades in the phylogenetic analysis, we used these clades to analyse patterns of desaturase evolution in social Hymenoptera. We reconstructed a phylogeny of social Hymenoptera desaturase sequences and selected nine groups, either large clades with good bootstrap support, or clades constituted by species-specific multiple duplications (Figures 3 and 4) on which selection analyses were performed. We used only the ant sequences (from the eight different ant species) for these analyses, the non-ant sequences being background branches. We first ran model M0 assuming the same background *ω* for all foreground branches of the tree to get *dS* estimates for all clades. The nine clades had *dS*<1 and were retained for subsequent selection analyses (*dS* (clade1) = 0.54; *dS* (clade2) = 0.22; *dS* (clade 3 = 0.20; *dS* (clade4) = 0.19; *dS* (clade 5) = 0.19; *dS* (clade 6) = 0.23; *dS* (clade 7) = 0.21; *dS* (clade 8) = 0.38; *dS* (clade 9) = 0.54).

To test whether these clades were under different selective pressures from one another, we compared the branch-model M2 which estimated a different *ω* for each clade, with an M2 model which estimated a single *ω* for all foreground branches. *ω* ratios were overall low, as expected for conserved proteins under purifying selection. However, we could detect a significant variation in *ω* among clades (p<0.001; Table 2). Clades 2, 4, 5 and 8 had *ω* estimates greater than the background *ω_0_*, which could mean that they evolved under more relaxed constraints or experienced more positive selection than the other clades. Clades 6 and 9 had *ω* estimates close to the background but included duplications we wanted to investigate. These six clades were thus chosen for site analyses. In contrast, clade 7 had an estimated *ω* close to the background *ω_0_* and was a single copy clade, and clades 1 and 3 had very low *ω* estimates and no duplications.

**Table 2:** Likelihood values and estimated parameters for branch-models in the Δ9 desaturase gene family of Hymenoptera.

Model	*L*	*k (ts/tv)*	*ω0*	*ω1*	*ω2*	*ω3*	*ω4*	*ω5*	*ω6*	*ω7*	*ω8*	*ω9*	LRT
M2	−46770.01	2.86	0.135	0.17	0.26	0.07	0.25	0.36	0.17	0.09	0.28	0.15	346***
M2 null	−49934.39	2.84	0.132	0.22	*ω1*	*ω1*	*ω1*	*ω1*	*ω1*	*ω1*	*ω1*	*ω1*	

*L* is the log of the likelihood, *k* the transition on transversion ratio, *ω* the ratio of non-synonymous to synonymous substitution rates;

*** significant at the 0.001% level. The M2 null model assumes the same *ω_1_* for all foreground branches.

Tests of site models were not significant for clades 2, 6, 8 and 9. Results of site models for the remaining clades are shown in Table 3. The M8 versus M8a comparison was significant for clade 4 (p<0.05). For clade 5, corresponding to the large ant-specific clade of the tree on Figure 2, both the M2a versus M1a and the M8a versus M8 tests were significant (p<0.05 and p<0.01, respectively). After correction for multiple tests, only the M8a versus M8 test for the clade 5 remained significant. A branch-site analysis on this clade showed that the expansion specific to the species *A. cephalotes* and *A. echinatior* was affected by positive selection (Table 3).

**Table 3:** Likelihood Ratio Tests for site and branch-site models in some clades of the Δ9 desaturase gene family.

Clades		2Δl^b^			Estimated parametersunder M8	Sites underpositive selection^c^
	*n* a	M1a vs M2a	M8 vs M8a	A vs A null		
clade 4	8	ns	8.66*	–	*p1* = 0.007 (*ω = *7.53)	260 V
clade 5	21	8.81*	11.5**	–	*p1* = 0.055 (*ω = *1.76)	178 K, 295 G, 300 N, 304 M
clade 5a	21			10.05*		

M1a versus M2a and M8 versus M8a comparisons are tests for site models. A versus A null is a test of branch-site selection.

* significant at 0.05 level.

** significant at 0.01 level.

a Number of sequences in the data set.

b Twice the logarithm of likelihood ratio.

c Positive selection sites estimated under M8 model by BEB approach with PPs >95% are listed.

As a comparison, Keays et al [43] analysed the evolution of codon divergence in genes of the *Drosophila* desaturase family. They found novel species-specific duplications in the genomes of several species of the *Drosophila* group. PAML analyses showed that sequences evolved under strong purifying selection overall, but that some duplicated copies evolved under positive selection, associated with changes in sex-specific expression for one of them. Knipple et al [84] found that desaturases in Lepidoptera evolved under strong purifying selection. They found six desaturase loci, and inferred signatures of positive selection in a specific lineage, but using the M8/M7 test, which has been shown to lead to many false positive [76]. Both studies show that putative selected sites are situated in terminal parts of the protein, outside the catalytic site and more likely to be involved in interaction with other proteins and/or in dimerisation. In our case, the putative selected sites are near the C-terminal part of the protein (Table 3), in accordance with the previous studies. Overall, these results support a pattern of evolution of desaturases in insects where birth-and-death processes play a large role (e.g. [85]), some duplicated copies being the targets of positive selection ([43], this study).

### Conclusions

The transcriptome dataset we obtained with 454 sequencing on a pooled sample of workers from 70 *F. exsecta* Finnish colonies represents the first large-scale genomic data available for the ant genus *Formica*. In addition, this study provides some general guidelines for *de novo* transcriptome assembly that should be useful for future transcriptome sequencing projects. Having sequenced a normalised cDNA library, our attention in this study focused on sequence variation. Deeper sequencing experiments and specific transcriptomics studies will be needed to complement this first large-scale dataset, in order to gain insights into patterns of gene expression in *Formica* ants.

One important goal has been achieved here: this first transcriptome reference sequence provides sequence and polymorphism data that will allow researchers working on *Formica* ants to develop studies to tackle the genetic basis of eusocial phenotypes. In particular, it will be useful for many genetic applications such as SNP genotyping, quantitative trait locus (QTL) analysis or genetic mapping which will be key to identify key genetic changes underlying important adaptations studied in *F. exsecta* and other *Formica* ant species, such as polymorphic social organisation and social parasitism.

The Δ9 desaturase gene family is an important multigene family involved in chemical communication in insects and which could play a major role in the evolution of sociality in social insects. Our analysis of these candidate genes in the *F. exsecta* transcriptome dataset identified a number of Δ9 desaturase genes but did not reveal any large gene family expansion in this species. Whole-genome sequencing data would be needed to confirm this result. However, the few expressed desaturase genes identified in *F. exsecta* transcriptome might play a key role in the regulation of CHC profiles, if each desaturase is involved in the final production of different length Z9-alkenes. An analysis of polymorphism and expression variation of these genes among different *F. exsecta* colonies will be required to further investigate this hypothesis and to gain insights into the genetic basis of nest mate recognition in this model species. For example, sequence data for desaturase and other candidate genes such as elongase or lipophorin genes could be used to monitor changes in levels of expression at these candidate genes associated with changes in CHC profile. Beyond sequence and expression variation, it would also be of interest to investigate copy number variation at desaturase loci as a potential source of genetic variation underlying chemical signaling among individuals, castes or populations. More generally, the large desaturase gene expansions revealed by our large-scale phylogenetic analysis in social Hymenoptera and the potential signature of positive selection on these genes suggest a very dynamic evolution of these genes in social insect genomes which could facilitate adaptation to sociality. Taking advantage of this transcriptome dataset, it will also be possible to apply these candidate gene approaches to other phenotypes studied in *Formica* ants, using findings in other ant taxa to provide predictions. For example, in the ant *Solenopsis invicta*, the number of queens is determined by a “social chromosome” [86], a large non-recombining region that includes the single gene, *Gp-9*, which is believed to code for an odorant-binding protein [87] but occurs widely throughout the body [88]. *Gp-9* was thought to be responsible for the determination of queen number, but recent evidence would tend to show that this is not the case [88], [89].

## Supporting Information

Figure S1 **Distribution of contig length in the** ***Formica exsecta de novo*** **transcriptome assembly.**
(TIF)

Figure S2 **Analysis of polymorphism in the transcriptome of** ***Formica exsecta.*** A: Number of SNPs in relation with contig length. B: Distribution of the number of SNPs per contig.
(TIF)

Table S1 **Accession number for all desaturase sequences used in this study.**
(XLS)

Table S2 **Comparison between** ***MIRA 3.0*** **and** ***Newbler 2.6.*** Basic metrics for *Newbler* and *MIRA* assemblies with varying minimum percent identity and minimum overlap length.
(DOC)

Table S3 **Effect of the option “-** ***urt*** **” on** ***Newbler 2.6*** **assembly.** This option is dedicated to improve the assembly in low coverage parts of the transcriptome.
(DOC)

Table S4 **Assessment of assembly quality on a set of test transcripts (35 test genes).** A – Contiguity of *MIRA 3.0* and *Newbler 2.6* assemblies with varying assembly parameters (minimum percent identity and minimum overlap length). For *Newbler 2.6* assemblies, the option “-*urt*” is on. B – Contiguity and completeness (standard deviation in brackets) for the best *Newbler 2.6* and *MIRA 3.0* assemblies. Best assemblies were determined based on basic metrics and coverage of test transcripts.
(DOC)

File S1 **cDNA and protein sequence information for all desaturase genes annotated in** ***Formica exsecta*** **transcriptome.**
(TXT)
