# Supplementary figures and images for: Transcriptome Characterisation of the Ant Formica exsecta with New Insights into the Evolution of Desaturase Genes in Social Hymenoptera
Source: PLoS One. 2013 Jul 12;8(7):e68200. doi: 10.1371/journal.pone.0068200 (PMC3709892; doi:10.1371/journal.pone.0068200)

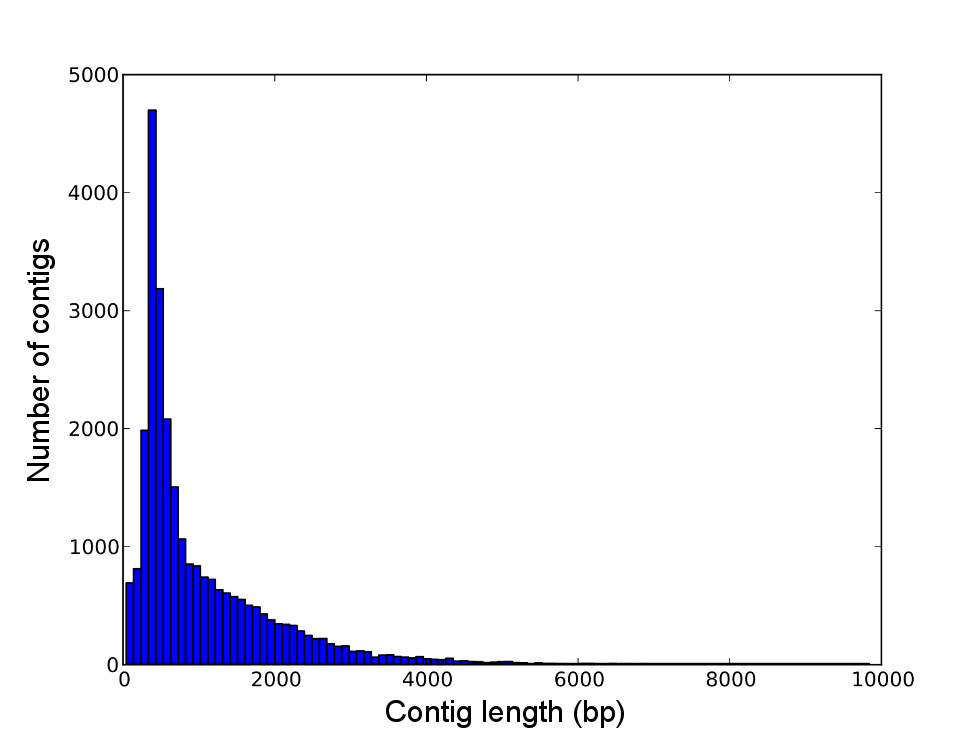

Supplement: Figure S1 — Distribution of contig length in the Formica exsecta de novo transcriptome assembly. (TIF) [file pone.0068200.s001.tif]

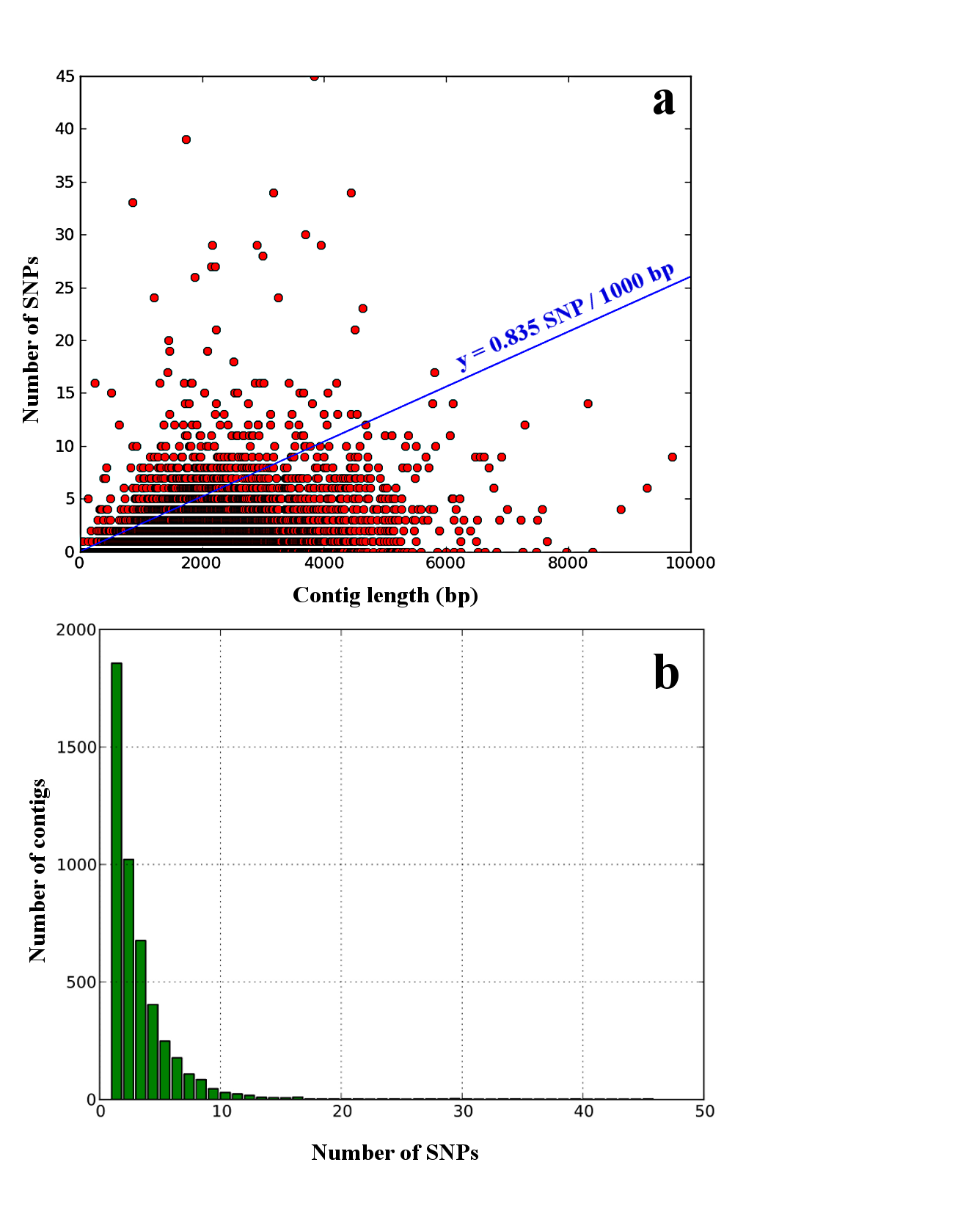

Supplement: Figure S2 — Analysis of polymorphism in the transcriptome of Formica exsecta. A: Number of SNPs in relation with contig length. B: Distribution of the number of SNPs per contig. (TIF) [file pone.0068200.s002.tif]
