# Supplementary material for: Transcriptome Characterisation of the Ant Formica exsecta with New Insights into the Evolution of Desaturase Genes in Social Hymenoptera
Source: PLoS One. 2013 Jul 12;8(7):e68200. doi: 10.1371/journal.pone.0068200 (PMC3709892; doi:10.1371/journal.pone.0068200)
Supplement: Table S2 — Comparison between MIRA 3.0 and Newbler 2.6. Basic metrics for Newbler and MIRA assemblies with varying minimum percent identity and minimum overlap length. (DOC) [file pone.0068200.s004.doc]

**Table S2**: Comparison between *MIRA 3.0* and *Newbler 2.6.* Basic metrics for *Newbler* and *MIRA* assemblies with varying minimum percent identity and minimum overlap length.

|  | ***MIRA 3.0*** | | | | ***Newbler 2.6*** | | | |
| --- | --- | --- | --- | --- | --- | --- | --- | --- |
| Minimum identity (%) | 90 | 90 | 85 | 95 | 90 | 90 | 85 | 95 |
| Overlap length (bp) | 20 | 40 | 20 | 40 | 40 | 60 | 40 | 60 |
| Number of contigs | 32,929 | 32,982 | 29,087 | 37,375 | 17,955 | 17,921 | 17,981 | 17,802 |
| Total length (Mb) | 26.6 | 26.2 | 26.1 | 25.3 | 1.60 | 1.60 | 1.60 | 1.60 |
| Longest contig (bp) | 6,047 | 5,657 | 6,984 | 4,125 | 10,327 | 10,325 | 10,326 | 10,324 |
| Average contig length (bp) | 809 | 797 | 895 | 679 | 893 | 890 | 892 | 892 |
| Number of singletons | 20,067 | 19,979 | 5,238 | 95,794 | 60,557 | 84,362 | 60,563 | 89,344 |
