# Supplementary material for: Transcriptome Characterisation of the Ant Formica exsecta with New Insights into the Evolution of Desaturase Genes in Social Hymenoptera
Source: PLoS One. 2013 Jul 12;8(7):e68200. doi: 10.1371/journal.pone.0068200 (PMC3709892; doi:10.1371/journal.pone.0068200)
Supplement: Table S3 — Effect of the option “- urt ” on Newbler 2.6 assembly. This option is dedicated to improve the assembly in low coverage parts of the transcriptome. (DOC) [file pone.0068200.s005.doc]

**Table S3**: Effect of the option “-*urt*” on *Newbler 2.6* assembly. This option is dedicated to improve the assembly in low coverage parts of the transcriptome.

|  | Average contig length (bp) | Number of contigs |
| --- | --- | --- |
| “urt” option off | 893 | 17,955 |
| “urt” option on | 722 | 32,576 |
