# Supplementary material for: Transcriptome Characterisation of the Ant Formica exsecta with New Insights into the Evolution of Desaturase Genes in Social Hymenoptera
Source: PLoS One. 2013 Jul 12;8(7):e68200. doi: 10.1371/journal.pone.0068200 (PMC3709892; doi:10.1371/journal.pone.0068200)
Supplement: Table S4 — Assessment of assembly quality on a set of test transcripts (35 test genes). A – Contiguity of MIRA 3.0 and Newbler 2.6 assemblies with varying assembly parameters (minimum percent identity and minimum overlap length). For Newbler 2.6 assemblies, the option “-urt” is on. B – Contiguity and completeness (standard deviation in brackets) for the best Newbler 2.6 and MIRA 3.0 assemblies. Best assemblies were determined based on basic metrics and coverage of test transcripts. (DOC) [file pone.0068200.s006.doc]

**Table S4**: Assessment of assembly quality on a set of test transcripts (35 test genes). A – Contiguity of *MIRA 3.0* and *Newbler* *2.6* assemblies with varying assembly parameters (minimum percent identity and minimum overlap length). For *Newbler* *2.6* assemblies, the option “-*urt*” is on. B – Contiguity and completeness (standard deviation in brackets) for the best *Newbler* *2.6* and *MIRA 3.0* assemblies. Best assemblies were determined based on basic metrics and coverage of test transcripts.

| **A** | *MIRA 3.0* | | | | | | *Newbler 2.6* | | | |
| --- | --- | --- | --- | --- | --- | --- | --- | --- | --- | --- |
| Assembly parameters (minimum percent identity - minimum overlap length | 85 - 20 | 85 - 40 | 90 - 20 | 90 - 40 | 95 - 20 | 95 - 40 | 85 - 40 | 90 - 40 | 95 - 40 | 90 - 40 + *uclust* |
| Contiguity (%) | 67.9 | 55.5 | 64 | 65.3 | 58.5 | 55.5 | 72.3 | 74.4 | 72.0 | 74.4 |

| **B** | *MIRA 3.0* | *Newbler 2.6* |
| --- | --- | --- |
| Completeness (%) | 81.0 (3.17) | 84.4 (2.72) |
| Contiguity (%) | 65.3 (3.66) | 74.4 (3.29) |
